# Osmolytes Contribute to pH Homeostasis of *Escherichia coli*

**DOI:** 10.1371/journal.pone.0010078

**Published:** 2010-04-08

**Authors:** Ryan D. Kitko, Jessica C. Wilks, Gian M. Garduque, Joan L. Slonczewski

**Affiliations:** Department of Biology, Kenyon College, Gambier, Ohio, United States of America; Charité-Universitätsmedizin Berlin, Germany

## Abstract

**Background:**

Cytoplasmic pH homeostasis in *Escherichia coli* includes numerous mechanisms involving pH-dependent catabolism and ion fluxes. An important contributor is transmembrane K^+^ flux, but the actual basis of K^+^ compensation for pH stress remains unclear. Osmoprotection could mediate the pH protection afforded by K^+^ and other osmolytes.

**Methods and Principal Findings:**

The cytoplasmic pH of *E. coli* K-12 strains was measured by GFPmut3 fluorimetry. The wild-type strain Frag1 was exposed to rapid external acidification by HCl addition. Recovery of cytoplasmic pH was enhanced equally by supplementation with NaCl, KCl, proline, or sucrose. A triple mutant strain TK2420 defective for the Kdp, Trk and Kup K^+^ uptake systems requires exogenous K^+^ for steady-state pH homeostasis and for recovery from sudden acid shift. The K^+^ requirement however was partly compensated by supplementation with NaCl, choline chloride, proline, or sucrose. Thus, the K^+^ requirement was mediated in part by osmolarity, possibly by relieving osmotic stress which interacts with pH stress. The rapid addition of KCl to strain TK2420 suspended at external pH 5.6 caused a transient decrease in cytoplasmic pH, followed by slow recovery to an elevated steady-state pH. In the presence of 150 mM KCl, however, rapid addition of another 150 mM KCl caused a transient increase in cytoplasmic pH. These transient effects may arise from secondary K^+^ fluxes occurring through other transport processes in the TK2420 strain.

**Conclusions:**

Diverse osmolytes including NaCl, KCl, proline, or sucrose contribute to cytoplasmic pH homeostasis in *E. coli*, and increase the recovery from rapid acid shift. Osmolytes other than K^+^ restore partial pH homeostasis in a strain deleted for K^+^ transport.

## Introduction

The enteric neutralophile *E. coli* maintains a cytoplasmic pH within a narrow range, approximately pH 7.4 to 7.8, when grown over a large range of environmental pH from pH 5 to 9 [1]–[3]. When *E. coli* experiences rapid external acid shift, the cytoplasmic pH falls, then largely recovers in less than 1 min [2], [4]. No single mechanism appears to be essential for pH homeostasis [5]–[8]. The regulation of cytoplasmic pH during acid stress depends on catabolic acid consumption and ion transport [5]. The effect of osmolytes on the cytoplasmic pH is important to understanding the survival of food pathogens such as *E. coli* O157, whose survival in extreme acid is enhanced by high NaCl [9]. Both *E. coli* and *Salmonella enterica* show prolonged survival in model acidic food broths with NaCl concentrations up to 4% [10], [11].

K^+^ transport plays a role in pH homeostasis of *E. coli* and other bacteria, although the mechanism remains unclear [7], [12], [13]. *E. coli* has many K^+^ transport systems, of which the major systems for K^+^ uptake are Kdp, Trk, and Kup (formerly TrkD) [14], [15]. Both Trk and Kup activity appear to involve proton symport, requiring the proton-motive force (PMF) to energize transport under aerobic conditions [14], [16], [17]. At low pH during hyperosmotic stress, Kup transport of K^+^ corresponds to a 1∶1 reduction in H^+^ efflux, suggesting the coupling of K^+^ influx to H^+^ influx [16]. Under aerobic conditions, transport of K^+^ by the Trk system is driven by the PMF and binding of ATP has a regulatory role [17]. Under anaerobic conditions, however, Trk may form a complex with the F_0_F_1_ ATP synthase, which is proposed to act as an ATP-driven K^+^/H^+^ antiporter [18]. The electrogenic activity of all three systems is thought to drive proton extrusion to maintain electroneutrality [14].

K^+^ transport is one of a large number of mechanisms contributing to pH homeostasis, some of them constitutive, others under regulons or distributed control [5]. Many bacteria primarily use net potassium uptake to compensate for proton extrusion that establishes the PMF, thus alkalinizing the cytoplasm at low external pH [12], [19]. In *E. coli*, the ΔpH of potassium-depleted cells varies with the K^+^ concentration of resuspension medium [20]. Furthermore, the cytoplasmic pH of K^+^-depleted cells increases upon addition of excess K^+^ [21]. A strain defective for all three uptake systems (Kdp, Trk, and Kup) shows a decreased growth rate at low pH and low potassium concentrations and fails to maintain a near neutral cytoplasmic pH during growth at pH 5.9 with 100 mM K^+^; an addition of excess K^+^ restores pH homeostasis in the triple mutant [22]. Nevertheless, the triple mutant shows limited K^+^ uptake through so-called “illicit” transport (also known as TrkF activity) of potassium through multiple minor pathways, such as mutated forms of the mechanosensitive channel MscL and the oligopeptide transport permeases of the *opp* operon [23].

The role of Kdp and TrkA in pH homeostasis is complicated by their roles in osmoregulation [24]. Osmoregulation in *E. coli* involves at least fifteen different transport systems for potassium and small molecules such as betaine and proline. The Kdp and TrkA systems take up K^+^ to maintain a normal cytoplasmic concentration of approximately 200 mM, or to an increased level at higher external osmolarities. Osmotic upshift activates the high-K_M_ system Trk, enabling K^+^ uptake in cells at concentrations above 1 mM. Below 1 mM K^+^, Trk is progressively supplemented by the low-K_M_ system Kdp (K^+^ uptake at micromolar concentrations). The Trk system is expressed constitutively while Kdp expression is modulated in part by osmolarity, via a sensor kinase/response regulator whose mechanism remains unclear.

Thus, in *E. coli* the K^+^ concentration contributes both to osmoprotection and pH homeostasis. How these two stress responses are connected remains unclear. Here we report the measurement of cytoplasmic pH in *E. coli* K-12 Frag1 as well as strain TK2420, a triple mutant defective for all major potassium-uptake mechanisms, under varying conditions of ion concentration and osmolarity. The cytoplasmic pH was measured by GFP fluorimetry [4], a technique allowing observation of both steady-state adaptation and kinetic responses on a 4-second time scale.

## Results

### Cytoplasmic pH measurement of Frag1 and TK2420 with different osmolytes

We tested whether K^+^ contributes to steady-state pH homeostasis, and whether other ions or osmolytes could substitute for K^+^. For measurement of cytoplasmic pH, the wild-type strain *E. coli* K-12 Frag1 and the triple mutant TK2420 were each transformed with the pH-dependent GFPmut3b reporter plasmid pMMB1311. Fluorescence was measured, and cytoplasmic pH was calculated, as described under Materials and Methods.

**Figure 1:**
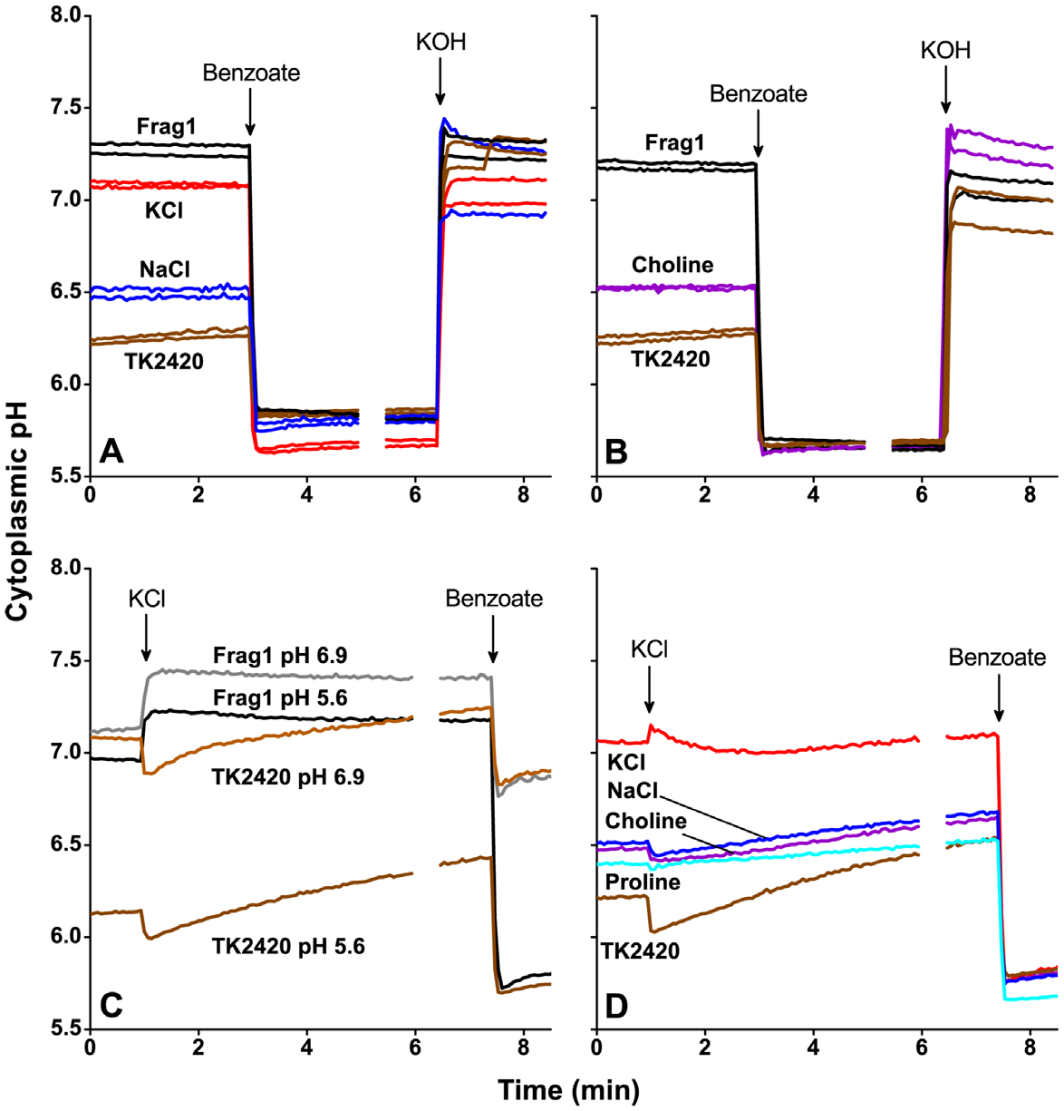
Effect of various osmolytes on cytoplasmic pH homeostasis. Fluorescence intensity was converted to cytoplasmic pH as described in the Materials and Methods with the addition of 30 mM sodium benzoate to collapse the ΔpH and addition of KOH to raise the pH for the second standard pH point. Each trace shown is a representative replicate of three biologically independent cultures; panels **A** and **B** show two replicate curves for each condition, indicating the range of minimal variability seen throughout our experiments. (**A**, **B**): *E. coli* K-12 strains Frag1 and TK2420 transformed with the GFPmut3b reporter plasmid (pMMB311) were resuspended in M63A medium (5 mM MES; pH 5.6) with different osmolytes. Each panel includes Frag1 (black) and TK2420 (dark brown) cultures in M63A medium that contains less than 10 mM of each K^+^ and Na^+^. The other conditions included TK2420 with an additional 300 mM KCl (red), TK2420 with an additional 300 mM NaCl (blue), and TK2420 with 150 mM choline chloride (violet). (**C**, **D**): Strains Frag1 and TK2420 transformed with the GFPmut3b reporter plasmid were resuspended in M63A and subjected to a rapid osmotic upshift with the addition of 150 mM KCl. Each panel includes a TK2420 (brown) culture in media that contain less than 10 mM of both K^+^ and Na^+^. The other conditions included (**C**) Frag1 at pH 6.9 (gray), Frag1 at pH 5.6 (black), and TK2420 at pH 6.9 (light brown); (**D**) all TK2420 at pH 5.6: 150 mM KCl (red), 150 mM NaCl (blue), 150 mM choline chloride (violet), and 300 mM proline (cyan). Addition of KOH is not shown.

Cytoplasmic pH was measured in the presence of various osmolytes (Fig. 1A, B). For each of these experiments, two representative replicates are shown for each condition; all subsequent figures show a single replicate, but the range of variation was comparable. Cultures were resuspended in M63A (5 mM MES) at pH 5.6, which includes less than 10 mM K^+^ ion. The cytoplasmic pH was measured for 3 min before collapsing the transmembrane pH difference with 30 mM sodium benzoate. Under these conditions, the parental strain Frag1 maintained a cytoplasmic pH of pH 7.16±0.03 (Fig. 1A). These pH values are consistent with our observations in an MC4100 background strain [4].

The TK2420 potassium transport-deficient strain, however, maintained a much smaller ΔpH and a cytoplasmic pH of pH 6.27±0.02 (Fig. 1A). When cultures of TK2420 were resuspended in media supplemented with an additional 300 mM KCl, the ability to maintain a large ΔpH comparable to that of the parent strain was largely restored (pH 7.04±0.04). Inclusion of an additional 300 mM NaCl (Fig. 1A), or of 150 mM choline chloride (Fig. 1B) led to partial restoration of cytoplasmic pH homeostasis (pH 6.5±0.02 and pH 6.54±0.01, respectively), though to a lesser degree than that seen with K^+^ supplementation.

### Cytoplasmic pH during rapid osmotic upshift

We tested the effect of rapid addition of KCl and other osmolytes on cytoplasmic pH of the parental strain and the triple mutant TK2420. GFPmut3b reporter strains of Frag1 (JLS0916) and TK2420 (JLS0917) were resuspended in M63A and subjected to a rapid addition of 150 mM KCl. The cytoplasmic pH was observed on a 4-s time scale (Fig. 1C, D). Cultures of Frag1 at both pH 5.6 and pH 6.9 experienced an immediate increase in cytoplasmic pH. The Frag1 cultures at pH 6.9 attained the higher constant cytoplasmic pH at pH 7.49±0.02, an increase from the initial cytoplasmic pH at pH 7.12±0.02, while cultures at pH 5.6 experienced an increase from pH 7.04±0.09 to pH 7.29±0.11.

Rapid KCl addition to strain TK2420 suspended at either pH 5.6 or pH 6.9 caused a sharp decrease in cytoplasmic pH of 0.15 to 0.18 pH units. The decrease was transient, followed by slow recovery over 5 min (Fig. 1C). When the initial medium already contained 150 mM KCl, however, a slight immediate increase in cytoplasmic pH occurred, followed by a decrease back to the starting pH. Cultures supplemented with 150 mM NaCl, 150 mM choline chloride, or 300 mM proline showed a small transient decrease in pH upon KCl addition, but not of the same magnitude of cultures suspended without any additional osmolytes; the pH decrease was followed by a slow recovery that brought the pH above the initial value (Fig. 1D). Cultures with NaCl, choline chloride, or proline present did not experience the same sharp decrease in cytoplasmic pH upon KCl addition, suggesting that osmolytes protect the cytoplasmic pH during perturbation.

### Rapid pH shift in MC4100

**Figure 2:**
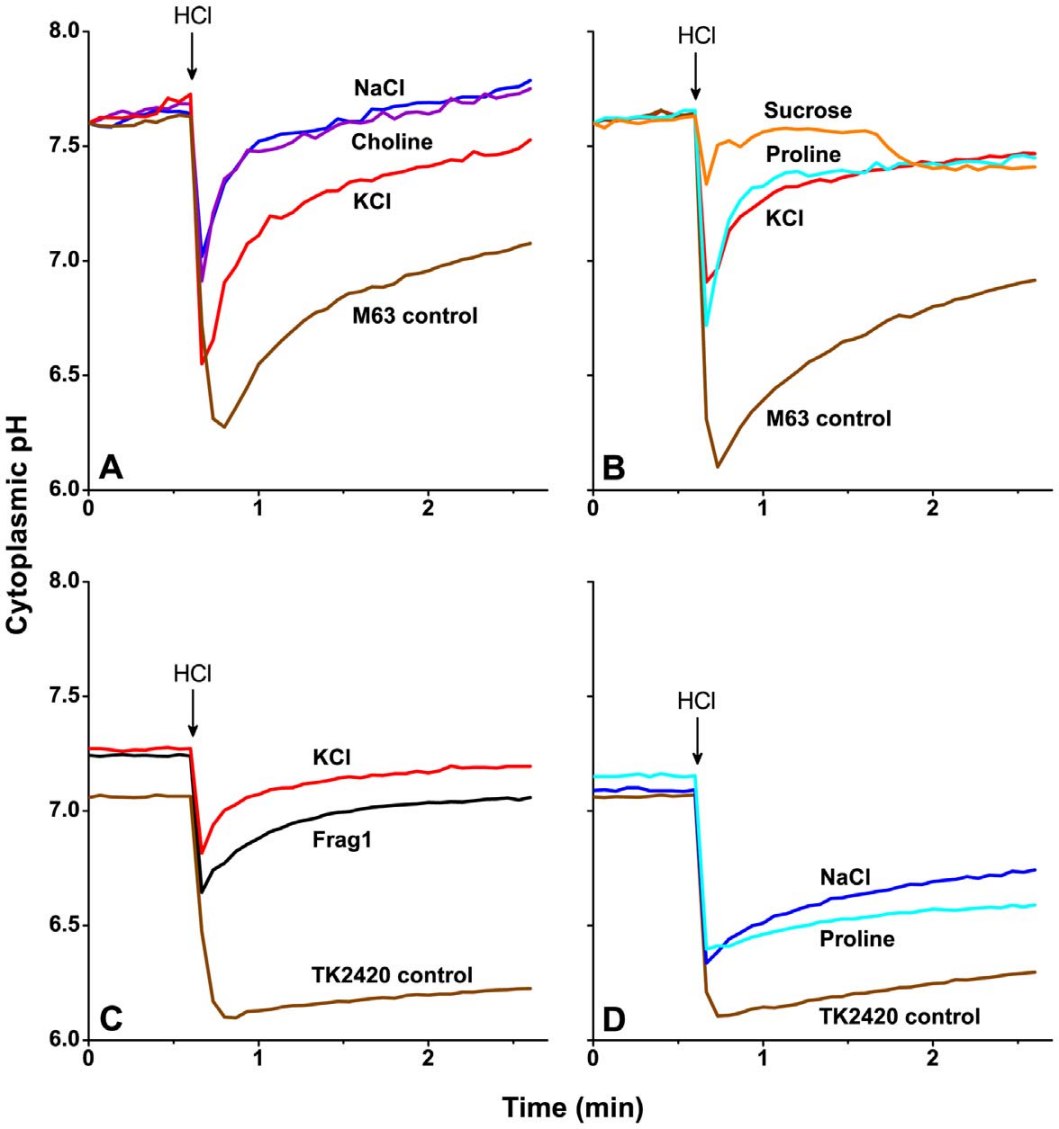
Effect of various osmolytes on cytoplasmic pH recovery after a rapid pH shift. Each trace shown is a representative replicate of three biologically independent cultures. Fluorescence intensity was converted to cytoplasmic pH as described in the Materials and Methods with the benzoate and KOH additions not shown. (**A**, **B**): *E. coli* strain MC4100AR Δ*tatABCDE* TorA-GFPmut3* was resuspended in M63A (5 mM HOMOPIPES; pH 7.5) and subjected to a pH shift to pH 5.5 with 8.5 mM HCl at 0.6 min (arrow). The media contained 100 mM NaCl (blue), 100 mM choline chloride (violet), 100 mM KCl (red), 200 mM sucrose (orange), 200 mM proline (cyan), or no added osmolyte (brown). (**C**, **D**): *E. coli* K-12 strains Frag1 and TK2420 transformed with the GFPmut3b reporter plasmid (pMMB311) were resuspended in M63A (5 mM MOPS, 5 mM MES; pH 7.0) with different osmolytes and subjected to an acid shift to pH 6.0 with approximately 10 mM HCl. Each panel includes Frag1 (black) and TK2420 (brown) cultures in M63A that contains less than 10 mM of both K^+^ and Na^+^. The other conditions included TK2420 with an additional 300 mM KCl (red line), TK2420 with an additional 300 mM NaCl (blue), and TK2420 with 300 mM proline (cyan).

We tested the effect of K^+^ and of other osmolytes on cytoplasmic pH recovery following a rapid acid shift. Cytoplasmic pH response was measured during rapid acidification of the external medium from pH 7.5 to pH 5.5, in the presence of several different osmolytes. The acid shift experiments were conducted with MC4100 Δ*tatABCDE* containing an arabinose-inducible TorA-GFPmut3* plasmid (Fig. 2A, B) [4]. Bacteria were resuspended in M63A buffered with 5 mM HOMOPIPES at pH 7.5, a medium estimated to contain less than 10 mM K^+^. The external pH was shifted to pH 5.5 by addition of 8.5 mM HCl.

The acid shift experiments were conducted under different ionic conditions: supplemented with 100 mM choline chloride or 100 mM NaCl (Fig. 2A) and 200 mM sucrose or 200 mM proline (Fig. 2B). Each set of experiments included three biological replicates without added solutes and three replicates with 100 mM KCl added. Cultures resuspended without additional osmolytes experienced an initial cytoplasmic pH drop to pH 6.07±0.05 with rapid recovery. The cytoplasmic pH of cultures resuspended with any solute (KCl, NaCl, proline, or sucrose) maintained a higher minimum pH during acid shock: 6.85±.08 for KCl; 6.93±0.05 for NaCl; 6.84±0.06 for proline; and 7.24±0.05 for sucrose. No significant difference was observed among the type of solute (one-way ANOVA: *F*(5, 12) = 2.01, *p* = 0.15). Similar results were obtained with inclusion of 50 mM K_2_SO_4_ or 100 mM K^+^ gluconate; thus, the anion made no difference (data not shown). The presence of an osmolyte also enhanced the overall pH recovery, allowing recovery to a higher level of cytoplasmic pH than observed in the unsupplemented M63A controls (Fig. 2A, B). These results indicate that in a wild type strain, pH recovery following rapid acid shift depends on osmolarity, rather than specifically on K^+^ concentration.

### Rapid pH shift in TK2420

We tested the effect of a rapid acid shift on the cytoplasmic pH recovery in the triple mutant strain TK2420. GFPmut3b reporter strains of Frag1 and TK2420 were resuspended in M63A (5 mM MES, 5 mM MOPS) at pH 7.0 and subjected to an acid shift to pH 6.0, using approximately 10 mM HCl (Fig. 2C). Cytoplasmic pH recovery of the strains was observed after rapid acidification of the medium. With minimal potassium and sodium present, Frag1 exhibited the normal cytoplasmic pH recovery profile (Fig. 2C) as previously described [4]. The K^+^-uptake triple mutant TK2420 in M63A supplemented with 150 mM KCl exhibited the same rapid recovery as the parental strain (Fig. 2C). Without supplelmental KCl, however, the cytoplasmic pH of TK2420 dropped nearly to the external pH with minimal recovery, though still maintaining a small ΔpH (Fig. 2C, D). The cytoplasmic pH of TK2420 was partially restored in the presence of 300 mM NaCl and 300 mM proline (Fig. 2D), showing a more distinct recovery. Additionally, NaCl or proline prevented the cytoplasmic pH from dropping as low as the control TK2420 cultures without additional osmolytes. This result suggests that any osmolyte will offer partial protection against rapid acidification.

## Discussion

Cytoplasmic pH homeostasis and acid resistance in *E. coli* interact with numerous other kinds of stress, such as anaerobiosis, oxidative stress, and stationary phase [5]. Anaerobiosis, specific amino acids, and low pH co-induce amino-acid decarboxylases that generate polyamines [6], [25]. Stationary phase greatly enhances survival in extreme acid, including mechanisms mediated by RpoS [26], [27]. In addition, acid stress up-regulates enzymes and envelope proteins that protect the cell from oxidative stress and extracytoplasmic stress [28]–[30]. Acid resistance can be increased by high NaCl and sucrose concentrations [9]–[11]. A possible mechanism for the role of Na^+^ in acid protection may involve induction of the Gad acid resistance regulon [31]. Thus it is interesting to consider whether osmoprotection may contribute part of the observed effect of K^+^ on pH homeostasis.

Potassium transport has long been understood to play a role in pH homeostasis, possibly by storing energy to drive H^+^ efflux or influx, either by symport or antiport mechanisms. Our data shows that another important function of K^+^ in cytoplasmic pH homeostasis is osmoregulation. In wild-type cells the contributions of K^+^, Na^+^, and organic osmolytes to pH recovery appeared equivalent (Fig. 2A, B). The mechanism of the link between osmoregulation and pH remains unclear, but it may involve maintaining cell volume and stabilizing ion fluxes.

Exogenous KCl restores pH homeostasis to the triple K^+^ transport mutant strain TK2420 under steady-state conditions (Fig. 1A), during a rapid acid shift (Fig. 2C), and as reported previously for the triple mutant strain TK2401 [22]. Growth rates of triple mutant strains at low pH are also restored to rates near that of the parental strain when excess KCl is included in the growth medium (data not shown). Yet we found that partial pH homeostasis was restored to TK2420 by addition of NaCl (Fig. 1A) or choline chloride (Fig. 1B) at low pH. Osmolyte compensation was observed both for cells resuspended in the presence of an osmolyte (Fig. 1A, B) and for cell suspensions subjected to an osmotic upshift with the rapid addition of 150 mM KCl (Fig. 1C, D). These observations indicate that other osmolytes may compensate for some of the lost contribution of K^+^. The osmolyte compensation is seen both for steady-state pH homeostasis (Fig. 1) and for cytoplasmic pH recovery following external acid shift (Fig. 2).

The kinetics of pH response to osmolyte addition (Fig. 1C, D) show a rapid immediate effect on cytoplasmic pH, followed by a more gradual adaptation. The parental strain Frag1 achieved a slightly higher cytoplasmic pH (and ΔpH) in the presence of KCl at both pH 5.6 and pH 6.9. These data conform to the models presented for cytoplasmic pH increase upon osmotic upshift in wild type cultures already in the presence of some potassium [24], [32]. For the triple mutant, however, the cytoplasmic pH fell transiently for about 8 seconds when KCl is added and then recovers slowly to a level higher than the original. Osmotic upshifts similar to this rapid KCl addition are associated with an immediate and significant reduction in cytoplasmic volume, followed by slow recovery that is similar in duration to the cytoplasmic pH recovery in the triple mutant strain shown in Fig. 1C [33]. The volume reduction increases cytoplasmic solute concentrations, possibly increasing the overall contribution of acidic components to a reduction in cytoplasmic pH. McLaggan *et al.* reported a reduction in both cytoplasmic pH and volume upon addition of 0.9 M glucose to potassium-depleted cultures [34], suggesting a coupling between cytoplasmic volume and pH in the absence of potassium.

KCl addition to TK2420 cultures pre-supplemented with 150 mM KCl caused cytoplasmic pH to rise transiently, then fall (Fig. 1D). These observations might be explained based on the kinetics of the poorly understood “illicit” transport processes that can mediate some K^+^ flux in a triple mutant strain [23]. In absence of K^+^, an osmotic upshift with KCl may increase flux through the secondary K^+^ transport pathways postulated by Buurman *et al.* [23]. Potassium cotransport with a proton could explain the rapid decrease in cytoplasmic pH seen in the minimal K^+^ TK2420 cultures (Fig. 1D). When cytoplasmic K^+^ attained a sufficient internal concentration through this transport activity, cytoplasmic pH began to recover as K^+^ drove proton extrusion. The transient increase in cytoplasmic pH observed upon osmotic upshift with KCl to TK2420 cultures where KCl was already present could be explained by the cotransport of potassium and protons out of the cell, causing the transient increase in cytoplasmic pH observed in Fig. 1D. The secondary K^+^ transport activity reported in Ref. [23] showed a strong dependence on external pH.

We have shown directly that various osmolytes, including K^+^, Na^+^, choline chloride, and proline can elevate cytoplasmic pH under acid stress, both in wild-type *E. coli* K-12 strains and in the K^+^ deficient triple mutant. Our data may shed light on earlier results that concluded Na^+^ enhances survival at pH 2.5 more strongly than K^+^ [31], whereas exogenous K^+^ enhances pH homeostasis during growth in moderately acidic medium where growth is still possible [20], [21]. Osmotic enhancement of pH homeostasis may play a role in the survival of pathogenic strains of *E. coli* and *Salmonella enterica* in acidic food products, where elevated NaCl concentration decreases the bacteriocidal effect of low pH [9]–[11].

## Materials and Methods

### Cytoplasmic pH measurements using GFPmut3b

*E. coli* strains Frag1 and TK2420 ((Δ*kdpFAB*)5 Δ(*trkA*-*mscL*') *trkD1*), kindly supplied by Wolfgang Epstein, were transformed with pMMB1311 (GFPmut3b) [35], creating strains JLS0916 and JLS0917, respectively. The strains were cultured overnight in Luria broth (LB) buffered with 50 mM 3-(N-morpholino)propanesulfonic acid (MOPS) adjusted to pH 7.5 with NaOH and included only 5 mM KCl for minimal K^+^-carry over into the resuspension media. Overnight cultures were incubated for approximately 16–18 hours at 37°C with 50 µg/ml ampicillin to maintain the plasmid. The cultures were diluted 100-fold into the same LB medium as the overnight cultures in 250-ml baffled flasks rotating at 260 rpm in a 37°C shaker bath. Bacteria were cultured to approximately OD_600_ 0.4–0.5 and then centrifuged (5000 rpm, 25°C, 10 min) and resuspended in 3 ml buffered M63A medium (5 mM 2-(N-morpholino)ethanesulfonic acid (MES) for the steady-state and KCl additions; 5 mM MES and 5 mM MOPS for the pH shifts) with varying osmolyte concentrations added to experimental conditions, including KCl, NaCl, choline chloride, and proline. K^+^ and Na^+^ were kept at a minimum for control replicates at concentrations less than 10 mM. Cell suspension fluorimetry on a 4-s time scale was performed as previously described [4], [35]. After recording sufficient excitation spectra every 4 s, 30 mM sodium benzoate was added to collapse the ΔpH and provide the first point where directly measured external pH equaled the fluorescence intensity. The pH of the cell suspension did not change significantly when the benzoate was added. While the ΔpH remained collapsed, the pH was then raised with KOH for a second direct measurement of pH and fluorescence intensity. Signal intensity was converted to cytoplasmic pH values by interpolation from the slope of the line between the two known pH/fluorescence intensity points. For each condition, three biologically independent trials were performed. Each set of trials was conducted at least twice. Figures show one or two representative curves for each condition; results were highly consistent among replicates. Error stated in the text is standard error of the mean (SEM; n = 3); and where necessary significance of the results was assessed with a one-way analysis of variance (ANOVA).

Fluorimetry optimized for this reporter plasmid was performed as described previously [4], [35]. Excitation spectra of the cell suspensions were recorded using a Fluoromax-3 spectrofluorimeter (Horiba Jobin Yvon). For each measurement, a 3-ml sample was placed into a Starna Spectrosil quartz cuvette with a path length of 10 mm. The temperature of the chamber was adjusted to 30°C. GFPmut3b excitation was measured from 480 to 510 nm (2 nm slit width) with an emission wavelength of 545 nm (20 nm slit width). Data were corrected for changes in excitation intensity by dividing the spectrally corrected emission data by the spectrally corrected excitation intensity (Sc/Rc). Continuous excitation spectra were recorded every 4 s for up to 8.5 min.

### Cytoplasmic pH measurements using TorA-GFPmut3*

*E. coli* strain MC4100 Δ*tatABCDE* containing a TorA-GFPmut3* plasmid [4] was cultured overnight in LBK (10 g tryptone, 5 g yeast extract, 7.45 g of KCl per liter) buffered with 50 mM homopiperazine-N, N'-bis-2-ethane-sulfonic acid (HOMOPIPES) at pH 7.5 and contained 50 µg/ml ampicillin. The overnight culture was diluted 1000-fold into pre-warmed 250-ml baffled flasks containing 10 ml of buffered LBK (20 mM HOMOPIPES, pH 7.5). In order to ensure plasmid selection and GFPmut3* expression, ampicillin (50 µg/ml) and L-arabinose (200 µg/ml) were added to the medium. Bacteria were cultured to an optical density at 600 nm (OD_600_) of 0.8–0.9 at 37°C in a shaker bath rotating at 250 rpm. For cytoplasmic pH measurement, the cultures were resuspended at OD_600_ 0.4 in buffered M63 medium [36] substituted with 0.2% casein hydrolysate (M63A) adjusted to pH 7.5 (5 mM HOMOPIPES). The buffering capacity of the media was sufficient to maintain the external pH for the duration of the fluorimetry. The osmotic strength of the suspension media depended on the experiment. All cultures were stored on ice until fluorimetry. The fluorimetry protocol is the same as described above for GFPmut3*. Continuous excitation spectra were obtained every 4 s for 2.6 min. After 0.6 min, 8.5 mM HCl was added to the cuvette to lower the pH from 7.5 to 5.5. At the end of each time-course, 40 mM sodium benzoate was added to collapse the ΔpH. Signal intensity was converted to pH units by interpolating between the benzoate signal intensity and the signal intensity before HCl addition as described previously [4]. Figures show one representative trial out of three independent cultures; all sets of trials were reproduced at least twice. Error stated in the text is SEM (n = 3).
